# Antibiotic Bowel Decontamination in Gastrointestinal Surgery—A Single-Center 20 Years’ Experience

**DOI:** 10.3389/fsurg.2022.874223

**Published:** 2022-05-16

**Authors:** Josefine Schardey, Thomas von Ahnen, Emily Schardey, Alina Kappenberger, Petra Zimmermann, Florian Kühn, Joachim Andrassy, Jens Werner, Helmut Arbogast, Ulrich Wirth

**Affiliations:** ^1^ Department of General, Visceral and Transplant Surgery, Ludwig-Maximilians-University Munich, Munich, Germany; ^2^ Institute for Surgical Research Oberbayern, Hausham, Germany; ^3^ Department for General, Visceral, Endocrine and Vascular Surgery, Krankenhaus Agatharied GmbH, Hausham, Germany

**Keywords:** antibiotic bowel decontamination, gastrointestinal surgery, anastomotic leakage, SDD, colorectal cancer, gastric cancer

## Abstract

**Objective:**

Anastomotic leakage, surgical site infections, and other infectious complications are still common complications in gastrointestinal surgery. The concept of perioperative antibiotic bowel decontamination demonstrates beneficial effects in single randomized controlled trials (RCTs), but data from routine clinical use are still sparse. Our aim was to analyze the data from the routine clinical use of perioperative antibiotic bowel decontamination in gastrointestinal surgery.

**Methods:**

Based on 20 years’ experience, we performed a retrospective analysis of all cases in oncologic gastrointestinal surgery with the use of antibiotic bowel decontamination in gastric, sigmoid, and rectal cancer. Clinical data and perioperative outcomes were analyzed, especially regarding anastomotic leakage, surgical site infections, and other infectious complications.

**Results:**

A total of *n* = 477 cases of gastrointestinal surgery in gastric cancer (*n* = 80), sigmoid cancer (*n* = 168), and rectal cancer (*n* = 229) using a perioperative regimen of antibiotic bowel decontamination could be included in this analysis. Overall, anastomotic leakage occurred in 4.4% (2.5% gastric cancer, 3.0% sigmoid cancer, 6.1% rectal cancer) and surgical site infections in 9.6% (6.3% gastric cancer, 9.5% sigmoid cancer, 10.9% rectal cancer). The incidence of all infectious complications was 13.6% (12.5% gastric cancer, 11.3% sigmoid cancer, 15.7% rectal cancer). Mortality was low, with an overall rate of 1.1% (1.3% gastric cancer, 1.8% sigmoid cancer, 0.4% rectal cancer). Antibiotic decontamination was completed in 98.5%. No adverse effects of antibiotic bowel decontamination could be observed.

**Conclusion:**

Overall, in this large cohort, we can report low rates of surgery-related serious morbidity and mortality when perioperative antibiotic bowel decontamination is performed. The rates are lower than other clinical reports. In our clinical experience, the use of perioperative antibiotic bowel decontamination appears to improve patient safety and surgical outcomes during gastrointestinal oncologic procedures in a routine clinical setting.

## Introduction

Digestive tract surgery is associated with high rates of surgical site infections (SSIs) as well as other infectious complications (1–5) and major elevation of treatment costs (6). The rate is the highest in colorectal cancer surgery, where infectious complications affect up to 26% of patients (7–9). The most severe complication of digestive tract surgery however is anastomotic leakage (AL), with an incidence in colorectal resections ranging from 5% to 15% and an associated mortality rate of 6%–30% (10–13). The leakage rate of esophagojejunal anastomosis following total gastrectomy is reported to be between 4% and 15% in recent literature (14–16), and the mortality in case of AL reaches up to 60% (17). AL of upper and lower gastrointestinal tract surgery not only causes morbidity and postoperative mortality but also impairs long-term cancer survival (2, 18–21).

While the role of bacteria in the development of SSI is unquestioned, their role in the pathogenesis of AL is not well accepted (10, 22–24). Today however there is experimental and clinical evidence, indicating that microbiota is directly involved in the pathogenesis of intestinal AL (10, 23, 25, 26). In 1994, Schardey demonstrated that deliberate postoperative contamination of esophagointestinal anastomoses with virulent *Pseudomonas aeruginosa* in rats resulted in AL rates of 95% (24). A topical application of nonresorbable antibiotics administered perioperatively until the 10th postoperative day reduced bacterial counts by 95%, and no AL occurred (24). He modified the selective decontamination of the digestive tract regimen (SDD), originally reported by Stoutenbeek et al. for the prevention of pneumonia in ventilated patients, adding vancomycin for double antibiotic coverage of relevant germs (27).

In a clinical multicenter randomized controlled trial (RCT), for the first time, Schardey demonstrated a significant reduction of AL in patients using the modified SDD regimen in patients with total gastrectomy for topical decontamination in gastric cancer surgery (28). It is also noteworthy that the number of postoperative pneumonia decreased significantly, and treatment costs were reduced by about 20% (28, 29). In a further clinical RCT, this modified SDD regimen was used in patients undergoing (low) anterior resection for rectal cancer (30). There was a significant reduction of AL in treatment compared to the control group, with a cost reduction in the treatment group of up to 37% (30).

Nevertheless, the use of bowel decontamination in gastrointestinal surgery is not widespread in Europe or United States (31–33), despite reliable data are available from prospective studies, meta-analyses, and large clinical registry cohorts (34, 35). Currently, several randomized trials have recently been published on the role of perioperative antibiotic bowel decontamination in colorectal surgery to prevent SSI, AL, and other infectious complications (9, 36, 37). However, only sparse data from the routine clinical use of decontamination in gastrointestinal surgery are available at present. Furthermore, there are other concepts in which antibiotic bowel decontamination is performed only preoperatively with or without combination with mechanical bowel preparation (38).

Based on the work by Schardey et al., there is 20 years’ experience in the routine use of antibiotic decontamination in nearly all patients undergoing gastric or colorectal surgery with primary anastomosis (28, 30, 39, 40). Especially patients with intestinal anastomoses to the esophagus, rectum, and anus have a higher risk for AL compared to other localizations in the gastrointestinal tract (10, 13, 28). The aim of this work is to analyze the routine clinical use of decontamination in surgery for gastric and colorectal cancer (CRC) concerning AL, SSI, and possible side effects over the available 20 years’ period in a single center.

## Materials and Methods

### Study Design

We designed a single-center retrospective cohort study including patients who received the preoperative and postoperative (modified) SDD regimen in upper and lower GI cancer surgery between 1999 and 2020 (on treatment) in an academic teaching hospital. The study was approved by the local review board (19-621 and 22-0013).

All elective procedures of gastric cancer surgery and of lower GI surgery for sigmoid and rectal cancer were analyzed. The hospital’s electronic database was used to identify all patients undergoing gastrectomy for gastric cancer as well as a sigmoid or rectal resection for CRC with primary anastomosis. In colorectal cancer surgery, cases without primary anastomosis (*Hartmann* procedure) or abdomino-perineal rectal amputations were excluded from the analysis. Overall, *n* = 477 cases met the selection criteria and received perioperative antibiotic bowel decontamination (SDD), with *n* = 80 cases of gastric, *n* = 168 cases of sigmoid, and *n* = 229 cases of rectal cancer.

### Antibiotic Decontamination (SDD) Regimen

An SDD regimen consisting of polymyxin B (100 mg), gentamicin (80 mg), and amphotericin B (500 mg) in sigmoid resections and a modified SDD regimen with additional use of vancomycin (125 mg) in gastric and rectal cancer surgery (PTVA) were used as previously described (28, 30). Patients without any perioperative SDD treatment were excluded from analysis (on treatment). The medication was administered four times daily. Amphotericin B was administered 30 min after the antibiotics. SDD application was usually started in the evening before surgery and continued every 6 h until the 7th postoperative day. For patients undergoing gastrectomy, the antimicrobial agents were dissolved in distilled water and administered as a solution per os (28). Patients with surgery for sigmoid or rectal cancer took these antibiotics as capsules per os (40). If a diverting stoma was created, an unblocked Foley catheter was placed transanally after the creation of the anastomosis, and antibiotics were then applied topically *via* the catheter dissolved in distilled water (30). The compliance of application as well as the completeness of decontamination regimen was controlled by evaluation of all patient files. All patients undergoing rectal cancer surgery received additional mechanical bowel preparation; the patient with sigmoid cancer had mild laxative therapy only. Gastric cancer patients received no additional bowel preparation.

Rectal cancer surgery was performed according to current technical standards, especially the total mesorectal excision (TME) technique was used for all low anterior rectal resections. Circular double-row staplers (Ethicon Circular Stapler, Ethicon Endo-Surgery, Johnson and Johnson, USA) were used for anastomoses in different sizes in gastric, sigmoid, and rectal cancer surgery. Intraoperative routine leak testing with a methylene blue solution was performed in every case. The extent of resection in gastric cancer patients depended on preoperative pathhistologic report and localization of the tumor according to medical evidence and national guidelines (41, 42). In all cases, a D2 lymphadenectomy was performed (43).

### Outcome Measures

Perioperative data (extent and type of surgery: subtotal/total/transhiatal extended gastrectomy, sigmoid resection, (low) anterior rectal resection [(L)AR] and multivisceral resection, use of minimally invasive surgery (MIC), TNM stage and UICC classification, all perioperative 30 day complications like infectious complications (AL, SSIs, urinary tract or pulmonary infections), and general complications (myocardial infarction, stroke, mortality)) were documented as well as other demographic data. The Charlson comorbidity index was calculated for all patients (44). Perioperative complications were classified according to the Clavien–Dindo classification (45), and additionally, the Clavien–Dindo comprehensive complication index (CCI) was calculated (46). Laboratory values such as white blood cell count and C-reactive protein (CRP) were assessed perioperatively. Potential adverse events associated with the SDD/PTVA regimen were also examined. Multivisceral resection was defined as additional resection of the small bowel, liver, or urogenital tract.

As previously described (39), AL was defined and classified according to the recommendation of the International Study Group for Rectal Cancer (47). AL was usually diagnosed by endoscopy, CT scan, or relaparotomy. Due to the retrospective design, only cases with clinically apparent AL could be included.

The primary endpoint is the rate of AL. Secondary endpoints are rates of surgical site infections (SSIs), infectious complications, overall morbidity and mortality, and adverse events related to the SDD/PTVA regimen.

### Statistical Analysis

For statistical analysis, SPSS 28 (IBM) and Graph Pad Prism V7 (V7 (GraphPad Software, Inc.)) were used. We performed a descriptive evaluation of perioperative outcome since no comparison of groups was possible as all patients received SDD treatment. Comparative analysis of patients with or without above-mentioned complications was carried out. A correlation of the Charlson comorbidity index and other risk factors with perioperative outcome was performed and an ROC analysis of laboratory parameters with regard to infectious complications. Patient characteristics and perioperative data were summarized using descriptive statistics and calculation of mean values. For comparison between different groups, we used the Mann–Whitney *U*-test (MW) for non-normally distributed values and Student’s *t*-test for normally distributed values. The normal distribution of mean differences was tested with the Kolmogorov–Smirnov test. Fisher’s exact and *χ*^2^ tests were used to compare data between subgroups involving nominal or categorical data. *p* values <0.05 were considered statistically significant.

## Results

### Patient Characteristics

In total, 477 surgical procedures with primary anastomosis and perioperative SDD treatment were included. Patients’ characteristics are summarized in Table 1.

**Table 1:** Demographic and descriptive information about the patients’ cohort.

		Gastric cancer *N* (%)	Sigmoid cancer *N* (%)	Rectal cancer *N* (%)
*N*		80	168	229
Sex, female/male		43/37	91/77	92/137
Age (mean ± SD)		71.6 ± 10.4	67.9 ± 11.2	67.8 ± 18.8
MIC		0	74 (44.0)	42 (18.3)
UICC	0	1 (1.3)	2 (1.2)	13 (5.7)
	I(a)	26 (32.5)	40 (23.8)	60 (26.2)
	Ib	11 (13.8)		
	IIa	12 (15.0)	38 (22.6)	55 (24.0)
	IIb	4 (5.0)	1 (0.6)	2 (0.9)
	IIIa	3 (3.8)	6 (3.6)	10 (4.4)
	IIIb	7 (8.8)	30 (17.9)	32 (14.0)
	IIIc	3 (3.8)	17 (10.1)	18 (7.9)
	IV	13 (16.3)	34 (20.2)	39 (17)
Mean Charlson comorbidity index (mean ± SD)		6.2 ± 2.3	6.0 ± 2.4	5.8 ± 2.4
Decontamination completed		78 (97.5%)	165 (98.2%)	227 (99,1%)
Multivisceral resection		17 (21.3%)	33 (19.6%)	34 (14.8%)

Most of the patients underwent surgery for CRC (sigmoid cancer *n* = 168, rectal cancer *n* = 229), and most often LAR (39.1%; *n* = 187), sigmoid resection (32.8%; *n* = 156) and AR (11.3%; *n* = 54) were performed. In patients with gastric cancer (*n* = 80), total gastrectomy (55%; *n* = 44) and partial gastrectomy (45%; *n* = 36) were performed. In surgery for gastric cancer, all procedures were carried out using the conventional open technique, whereas in 44% (*n* = 74) of patients undergoing surgery for sigmoid cancer and in 18.3% (*n* = 42) of patients with rectal cancer, the procedures were performed using the minimal invasive surgical technique (MIC). Multivisceral resection was necessary in approximately 15%–20% of procedures independent of underlaying disease (Table 1).

#### CRC

Patients with CRC had a mean age of 67.9 ± 11.2 years and 67.8 ± 10.7 years for sigmoid and rectal cancer, respectively. The mean Charlson comorbidity index for patients with sigmoid cancer was 6.0 ± 2.4 and that for rectal cancer was 5.8 ± 2.4. Patients with sigmoid cancer were mostly classified as UICC III-IV with 51.8% of cases (*n* = 87) and 47.0% of cases UICC I-II (*n* = 79). In rectal cancer patients, 53.9% were classified as UICC (y0)I-II (*n* = 130) and 41.1% were classified as UICC III-IV (*n* = 99) (Table 1). Decontamination was completed in *n* = 165 (98.2%) cases in sigmoid and *n* = 227 (99.1%) cases in rectal cancer patients.

#### Gastric Cancer

For gastric cancer, patients were slightly older, with a mean age of 71.6 ± 10.4 years. The mean Charlson comorbidity index for patients with gastric cancer was 6.2 ± 2.3. The majority of patients with gastric cancer were classified UIC I-II in 67.5% (*n* = 54) and UICC III-IV in 32.5% (*n* = 26) (Table 1). The decontamination regimen was complete in *n* = 78 (97.5%) of gastric cancer patients and not completed in *n* = 2 cases (2.5%).

### Perioperative Outcome

Outcome parameters are summarized in Table 2 (separated for diagnosis) and Table 3 (separated for surgical procedures). The CCI was the highest with a mean of 17.06 ± 17.65 for gastric cancer, with 23.75% major morbidity Clavien–Dindo IIIa–V (*n* = 19). In 35% (*n* = 28), no complications were reported, and in 41.3% (*n* = 33), only minor complications (Clavien–Dindo I–II) occurred.

**Table 2:** Outcome parameters for different diagnoses.

Dindo–Clavien classification	Gastric cancer *n* (%)	Sigmoid cancer *n* (%)	Rectal cancer *n* (%)	*p*-value *χ*^2^
No complication	28 (35.0)	122 (72.6)	103 (45.0)	*p* < 0.001*
I	12 (15.0)	19 (11.3)	49 (21.4)	
II	21 (26.3)	7 (4.2)	32 (14.0)	
IIIa	7 (8.8)	2 (1.2)	7 (3.1)	
IIIb	8 (10.0)	14 (8.3)	27 (11.8)	
IVa	3 (3.8)	0	8 (3.5)	
IVb	0	1 (0.6)	2 (0.9)	
V/mortality	1 (1.3)	3 (1.8)	1 (0.4)	
Comprehensive complication index	17.06 ± 17.65	7.50 ± 17.42	14.06 ± 18.41	*p* < 0.001*
Stroke	0	1 (0.6)	1 (0.4)	*p* = 0.793
Myocardial infarction	1 (1.3)	2 (1.2)	1 (0.4)	*p* = 0.729
Infectious complication	10 (12.5)	19 (11.3)	36 (15.7)	*p* = 0.426
Anastomotic leakage	2 (2.5)	5 (3.0)	14 (6.1)	*p* = 0.312
Pneumonia	2 (2.5)	3 (1.8)	3 (1.3)	*p* = 0.768
SSI	5 (6.3)	16 (9.5)	25 (10.9)	*p* = 0.635
Type 1	4 (5.0)	11 (6.5)	13 (5.7)	
Type 2	0	4 (2.4)	8 (3.5)	
Type 3	1 (1.3%)	1 (0.6)	4 (1.7)	
Urinary tract infection	1 (1.3)	1 (0.6)	1 (0.4)	*p* = 0.729
In-hospital stay	27.6 ± 21.2	13.3 ± 10.4	17.6 ± 12.0	*p* < 0.001*

*Statistically significant (p-value < 0.05).

**Table 3:** Outcome parameters for the type of surgery.

Disease	Colorectal cancer (CRC)			Gastric cancer		*p* value *χ*^2^
Type of surgery	Low anterior rectal resection (%)	Anterior rectal resection (%)	Sigmoid resection (%)	Total gastrectomy (%)	Partial gastrectomy (%)	
*n*	187	54	156	44	36
Completeness of decontamination	185 (98.8)	54 (100)	153 (98.1)	43 (97.7)	35 (97.2)	*p* < 0.001*
Infectious complications	32 (17.1)	5 (9.3)	18 (11.5)	5 (11.4)	5 (13.9)	*p* = 0.466
AL	14 (7.5)	0	5 (3.2)	2 (4.5)	0	*p* = 0.131
SSI	22 (11.8)	4 (7.4%)	15 (9.6)	2 (4.5)	2 (5.6)	*p* = 0.384
I	11 (5.9)	2 (3.7)	11 (7.1)	2 (4.5)	2 (5.6)	
II	8 (4.3)	0	4 (2.6)	0	0	
III	3 (1.6)	2 (3.7)	0	0	1 (2.8%)	
Mortality	1 (0.5)	0	3 (1.9)	1 (2.3)	0	*p* = 0.522

*Statistically significant (p-value < 0.05).

For sigmoid cancer in 72.6% (*n* = 122), no complications occurred, whereas in 15.5% (*n* = 26), minor complications (Clavien–Dindo I–II) were reported. In 11.9% of cases (*n* = 20), major complications occurred (Clavien–Dindo IIIa–V). The mean CCI was 7.50 ± 17.42.

In rectal cancer, in 45.0% of cases (*n* = 103), no complication and in 35.4% minor complications (*n* = 81) were documented. In 19.7% (*n* = 45), major morbidity (Clavien–Dindo IIIa–V) occurred. The mean CCI was 14.06 ± 18.41. The distribution of complications according to the Clavien–Dindo classification was different between gastric, sigmoid, and rectal cancer (*χ*^2^: *p* < 0.001) as well as between different surgical procedures (*χ*^2^: *p* < 0.001).

CCI was different between different diagnoses (KW: *p* < 0.001) and between the surgical procedures (KW: *p* < 0.001). Both stroke and myocardial infarction occurred only in three cases of CRC patients and in one patient suffering from gastric cancer.

#### Anastomotic Leakage

AL occurred in a total of *n* = 21 cases and was most frequent in rectal cancer surgery (*n* = 14; 6.1%). Regarding the procedure, AL occurred only in LAR (*n* = 14) and not in AR (*n* = 0) procedures. Another *n* = 5 cases occurred in sigmoid resections (3.0%) and *n* = 2 in surgery for gastric cancer (2.5%) (Table 2).

In patients with gastric carcinoma, there was one AL classified as grade B and C. In patients with sigmoid carcinoma, all cases of AL required surgical therapy (grade C). In patients with rectal cancer, AL was classified as grade A (*n* = 2; 1.9%), grade B (*n* = 4; 1.7%), and grade C (*n* = 8; 3.5%), requiring surgical treatment. The mean time (range) to the diagnosis of AL was 17 days (13–20) in gastric cancer, 7.6 days (5–10) in sigmoid cancer, and 8.6 days (1–15) in patients with rectal cancer surgery.

There was no significant difference in rates of AL between groups regarding the type of surgical procedure (LAR, AR, sigmoid resection, total gastrectomy, subtotal gastrectomy; *χ*^2^: *p* = 0.064). Also, multivisceral resection was not associated with increased rates of AL (Fisher: *p* = 0.252). There was no difference in rates of AL in open vs. MIC surgery (Fisher: *p* = 0.404), and rates of AL were not higher if conversion to open surgery was necessary (Fisher: *p* = 0.835). AL significantly prolonged the in-hospital stay (MW: *p* < 0.001).

Patients with AL had a significantly higher Charlson comorbidity index (MW: *p* = 0.048) across all diagnoses. Age did not significantly differ between patients with and without AL (MW: *p* = 0.258).

#### Infectious Complications

Overall, none of the diagnoses (rectal, sigmoid, or gastric carcinoma) showed an increased rate of infectious complications in general compared to the others (*χ*^2^: *p* = 0.426). However, there was a nonsignificant trend toward fewer infectious complications with minimally invasive surgery (*χ*^2^: *p* = 0.071). In the case of conversion to open surgery, infectious complications did not occur more frequently (Fisher: *p* = 0.425).

Patients with infectious complications showed a significantly higher Charlson comorbidity index than patients without infectious complications (MW: *p* = 0.010). These patients were significantly older than patients without infectious complications (MW: *p* = 0.049). As expected, hospital stay was significantly prolonged in patients with infectious complications (MW: *p* < 0.001).

#### Surgical Site Infection

SSIs occurred in 6.3% of cases in gastrectomies. SSI grade I–III was reported in 9.5% of cases for sigmoid cancer surgery (*n* = 16) and in 10.9% of cases (*n* = 25) for rectal cancer surgery (Table 2).

SSIs were distributed equally between groups of gastric, sigmoid, and rectal cancer surgery (*χ*^2^: *p* = 0.635). Even for the different types of surgical procedures, the rates of SSI were not different (*χ*^2^: *p* = 0.384). The in-hospital stay of patients suffering from SSI was significantly longer (30.5 ± 15.3 days vs. 16.1 ± 13.4 days; MW: *p* < 0.001). The Charlson comorbidity index was significantly higher in patients with SSI (6.7 ± 2.8 vs. 5.8 ± 2.3; MW: *p* = 0.042).

There was no significant difference in rates of SSI for the use of minimally invasive surgery (*χ*^2^: *p* = 0.187), conversion to open surgery (*χ*^2^: *p* = 0.478), or multi-visceral resection (*χ*^2^: *p* = 0.234). There was no difference in the distribution of SSI in different UICC stages (*χ*^2^: *p* = 0.335). Completed decontamination had no significant impact on the rate of SSI (*χ*^2^: *p* = 0.767).

#### Mortality

In gastric cancer cohort, there was a mortality rate of 1.3% (*n* = 1), 1.8% (*n* = 3) in sigmoid cancer and 0.4% (*n* = 1) rectal cancer surgery. Overall, the distribution of mortality was equal between gastric, sigmoid, and rectal cancer (*χ*^2^: *p* = 0.419). Patients who eventually died had a significantly higher age (79.6 ± 8.7 vs. 68.34 ± 10.8 years; MW: *p* = 0.028) and Charlson comorbidity index (9.2 ± 1.3 vs. 5.87 ± 2.4; MW: *p* = 0.003) than patients without in-hospital mortality. Patients who died had a significantly longer in-hospital stay than those who survived (24.2 ± 4.0 vs. 17.7 ± 14.4 days; MW: *p* = 0.022). Mortality rates were not different between MIC and open surgery (Fisher: *p* = 0.647) or if conversion to open surgery was necessary (Fisher: *p* = 0.959). In cases of multivisceral resections, mortality was not increased (Fisher: *p* = 0.214). The distribution of mortality was not different for UICC stages (*χ*^2^: *p* = 0.836). Complete decontamination did not have a significant impact on mortality rates (*χ*^2^: *p* = 0.926).

In the gastric cancer cohort, there was one patient who died due to AL-related septic complications. In patients with sigmoid cancer, one patient with AL and wound healing disorder developed a status epilepticus and died from septic complications and another patient died due to septic complications following grade II SSI with progressive multiorgan failure and pneumonia after aspiration, respectively. One patient developed a rapid cancer progression and associated pulmonary complications and died from respiratory insufficiency. In rectal cancer surgery, only one patient died from AL-related septic complications. This patient refused the necessary surgical therapy for AL.

### Analysis for Risk Factors in Univariate Analysis

In univariate analysis, the Charlson comorbidity index and multivisceral resection had a significant impact on the incidence of infectious complications, SSI, and AL. Additionally, the UICC stage had a significant impact on infectious complications in general only. However, diagnosis, use of MIC surgery, and completeness of decontamination had no effect on the occurrence of infectious complications, SSI, and AL. The univariate analysis revealed no significant risk factors for mortality (Table 4).

**Table 4:** Univariate analysis for infectious complications, anastomotic leakage, SSI, and mortality (*p* values  < 0.05 are marked with an *).

		Df	Mean of squares	*F*	Sig.
Infectious complications (*R*^2 ^= 0.044; *p* < 0.001*)	Charlson comorbidity index	1	1.431	12.592	*p* < 0.001*
	UICC stage	1	0.689	6.060	*p* = 0.014*
	Multivisceral resection	1	0.567	4.988	*p* = 0.026*
	Completeness of decontamination	1	0.061	0.539	*p* = 0.463
	Diagnosis	1	0.249	2.193	*p* = 0.139
	MIC	1	0.293	2.575	*p* = 0.109
Anastomotic leakage (*R*^2 ^= 0.246; *p* = 0.001*)	Charlson comorbidity index	1	4.708	146.133	*p* < 0.001*
	UICC stage	1	0.025	0.765	*p* = 0.382
	Multivisceral resection	1	0.167	5.186	*p* = 0.023*
	Completeness of decontamination	1	0.058	1.806	*p* = 0.180
	Diagnosis	1	0.062	1.933	*p* = 0.165
	MIC	1	0.109	3.392	*p* = 0.066
Surgical site infections (*R*^2 ^= 0.044; *p* = 0.025*)	Charlson comorbidity index	1	0.968	3.918	*p* = 0.048*
	UICC stage	1	0.917	3.711	*p* = 0.055
	Multivisceral resection	1	1.198	4.851	*p* = 0.028*
	Completeness of decontamination	1	0.038	0.153	*p* = 0.696
	Diagnosis	1	0.785	3.176	*p* = 0.075
	MIC	1	0.438	1.772	*p* = 0.184
Mortality (R^2 ^= 0.023; *p* = 0.091)	Charlson comorbidity index	1	0.059	5.746	*p* = 0.017*
	UICC stage	1	0.003	0.306	*p* = 0.580
	Multivisceral resection	1	0.004	0.360	*p* = 0.549
	Completeness of decontamination	1	<0.001	0.017	*p* = 0.896
	Diagnosis	1	0.006	0.556	*p* = 0.456
	MIC	1	<0.001	0.007	*p* = 0.933

*Statistically significant (p-value < 0.05).

### Diagnosis of Infectious Complications and Anastomotic Leakage Based on CRP Values

**Figure 1:**
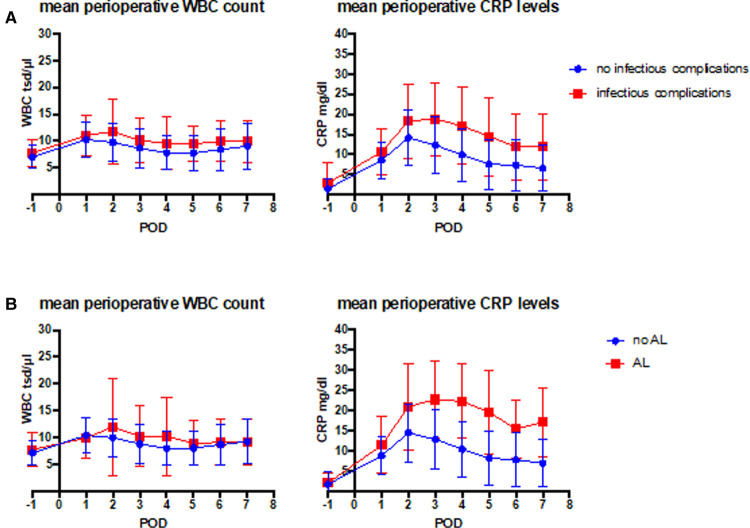
Laboratory values such as white blood cell count (WBC) and C-reactive protein (CRP) were assessed perioperatively from the day before surgery until the 7th postoperative day. (**A**) Comparison of the course of parameters between patients with (red) and without (blue) infectious complications. (**B**) Comparison of the course of the parameters between patients with (red) and without (blue) AL.

**Figure 2:**
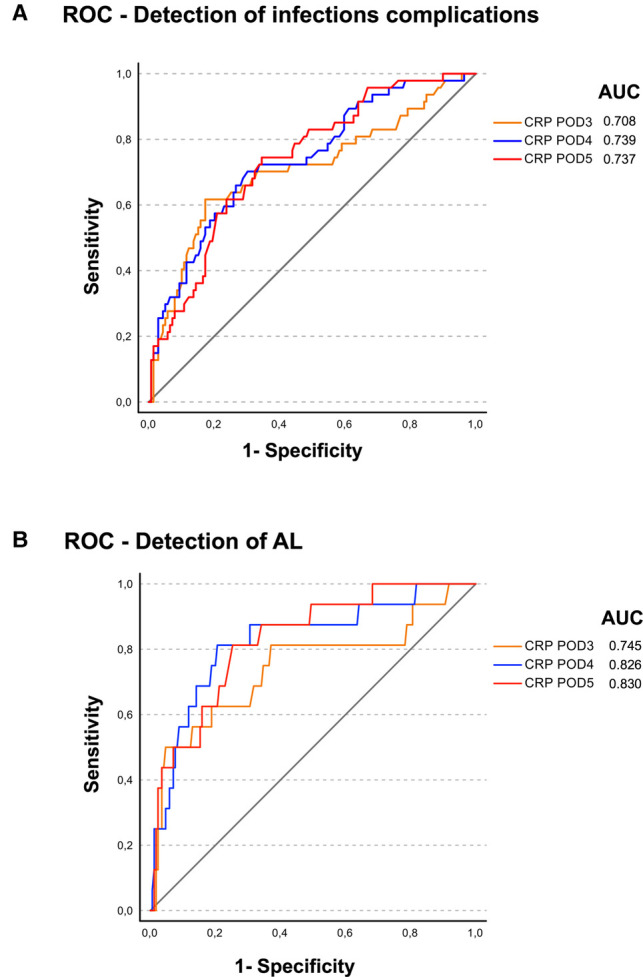
ROC analysis was perfomed for the postoperative CRP levels from postoperative days (POD) 3, 4, and 5 for (**A**) diagnosis of infectious complications and (**B**) diagnosis of AL with the area under curve (AUC) given in the figures.

Whereas the white blood cell count was not significantly different between patients with and without infectious complications or AL, the course of CRP values differed significantly (Figures 1A,B). ROC analysis showed that CRP values on days 4 and 5 discriminate not as good for diagnosis of infectious complications (AUC 0.739 and 0.737; Figure 2A) as for diagnosis of AL (AUC 0.826 and 0.830; Figure 2B) on days 4 and 5, respectively.

### Adverse Events Related to the SDD Regimen

Overall, in *n* = 2 patients with gastric cancer and *n* = 3 patients with sigmoid cancer, the SDD regimen was not completed due to nausea and possible intolerance, whereas in rectal cancer surgery in *n* = 2 cases, the catheter at the anastomotic site was dislocated or removed accidentally so that the SDD regimen could not be continued. Other side effects such as allergic reactions or intolerance did not occur.

Only in rectal cancer surgery there was one patient with clostridium difficile-associated diarrhea.

## Discussion

The routine clinical use of antibiotic decontamination in 477 patients with gastric, sigmoid, and rectal cancer surgery seems to be not only feasible but also successful with regard to the overall low rates of SSI, AL, and mortality. Certainly, the rates for AL and SSIs were higher in colorectal compared to gastric cancer surgery.

Although this is a retrospective study lacking a control group, the complication rates compare well with results achieved in double-blind RCTs for gastric (28) and rectal cancer surgery with the use of this SDD/PTVA regimen (30). Mortality rates were low and major complications were more frequent in gastric and rectal compared to sigmoid cancer patients. Patients with SSI, AL, and infectious complications in general and mortality had a significantly higher Charlson comorbidity index compared to patients without infectious complications. Hospital stay was significantly prolonged in these patients. Nearly all patients completed the perioperative antibiotic decontamination regimen, and no adverse events could be detected.

### Data on Gastric Cancer Surgery

In a recent review, AL of esophagointestinal anastomosis was reported with an incidence between 2.1% and 14.6% and associated mortality of up to 50% (48). Yoo et al. reported AL in 6.7% following curative resection of gastric cancer. Poor performance status and tumor localization were risk factors for leakage in the latter study (17). In our data, the Charlson comorbidity index was higher for patients with gastric cancer compared to CRC patients. Nonetheless, rates for infectious complications in our gastric cancer patients were low by any standard. In our patients, leaks occurred late in the postoperative course, which may be an effect of decontamination. In our experience, late leaks are less dangerous compared to leaks in the early postoperative course. Overall, only scarce data are available about the use of perioperative antibiotic decontamination in gastric cancer surgery. Scheufele et al. recently conducted a systematic review and meta-analysis of the current evidence for the role of SDD in RCTs of upper gastrointestinal tract surgery reporting a significant reduction in AL and postoperative pneumonia after total gastrectomy and esophagectomy using SDD regimens. These data support the routine use of the SDD regimen in gastrointestinal surgery (16).

### Data on Colorectal Cancer Surgery

The complication rates for sigmoid and rectal cancer surgery were much higher than our previously reported data on surgery for diverticulitis using the same SDD regimen (40). For rectal cancer surgery, reliable data about outcome measures without the use of antibiotic bowel decontamination are available from a large German cohort with rates for AL of 11.9% and overall in-hospital mortality of 2.1% (13).

Roos et al., based on their data of a systematic review, stated that a combination of perioperative SDD and perioperative intravenous antibiotic prophylaxis in elective gastrointestinal surgery reduces the rate of postoperative infections, including AL, compared with the use of intravenous antibiotics alone (5). These results have been confirmed by Abis et al., who analyzed the use of SDD in esophageal, gastric, and colorectal surgeries (49).

Results from recently published RCTs and meta-analyses report contrary outcomes of combined bowel preparation. The SELECT trial using a perioperative SDD regimen demonstrated a significant reduction of SSI but not AL (9). The MOBILE trial adding neomycin and metronidazole to mechanical bowel preparation preoperatively only failed to show a relevant difference in SSI or AL between the treatment and control groups (36). However, both trials included only a limited number of left-sided colonic and rectal resections. A meta-analysis recently published by Rollins et al. demonstrated a reduction in SSIs and AL mostly based on the included registry data. The meta-analysis of the RCTs alone did not show a relevant reduction of AL (38).

The available data lack consistency as different types of antibiotic regimens and durations of application are used as well as different types of surgical procedures are included (4, 9, 30, 36, 38, 49). Compared to the available RCTs and other data on the use of a perioperative SDD regimen in combination with mechanical bowel preparation, our analysis shows similar results regarding rates of infectious complication, SSI, and AL, despite the fact that most of these studies excluded UICC stage IV patients, whereas about 18% of UICC stage IV cases are included in our analysis (4, 5, 9, 30, 49). In summary, the relevant data on the use of the perioperative SDD regimen in colorectal surgery support the strategy of topical antibiotics in a reasoned combination (4, 9, 30, 39, 40).

### Effect of SDD on Multidrug Resistant Germs and Possible Side Effects

We are aware that there are increasing numbers of vancomycin-resistant Enterococci species (50), but published data on routine use of topical antibiotics like SDD in intensive care units show even a decrease in colonization of Enterococci species (51). Furthermore, there are reliable data on oral vancomycin, as it is widely used in Clostridioides difficile infections. Few antibiotic resistances to vancomycin occurred over time, with a treatment duration of 10 days or even longer (52–54). However, recent experimental data demonstrated a significant role of Enterococci species in the pathogenesis of AL (25, 55). Schardey et al. modified the SDD regimen for antibiotic decontamination by adding vancomycin to the usual SDD regimen. This modified SDD regimen seems to be much more efficacious as it covers a much larger spectrum of potentially pathogenic germs, most of them even twice, including Enterococci species, while these are not sufficiently covered by a conventional SDD regimen (24, 28, 30). On the other hand, the widespread use of antibiotics is a major concern regarding the development of antimicrobial resistance. Presently, the beneficial effect of topical antibiotics in the prevention of AL, in our opinion, outweighs the possible adverse side effects. In over 20 years of the use of these modified SDD regimens in gastrectomy and colorectal surgery, no adverse events regarding multidrug-resistant germs or other relevant side effects have been observed (30, 39, 40).

### Risk Factors for Anastomotic Leakage and Other Infectious Complications

In our data, we could detect some risk factors in univariate analysis like the Charlson comorbidity index and multivisceral resections for AL, SSI, and infectious complications in general. Other data already demonstrated male sex, obesity, neoadjuvant (radio)chemotherapy, an impaired preoperative physical and nutritional state or ASA ≥ 3 patients, smoking, UICC stage, and operative factors like level of anastomosis, surgeon volume, and not creating a diverting stoma in low anterior rectal resections as risk factors for AL (12, 13, 56). In our data, due to a limited number of events, no reliable analysis of risk factors for anastomotic leakage and other infectious complications despite the results of the univariate analysis has been possible.

Furthermore, our analysis shows that CRP levels on postoperative days 4 and 5, to some extent, seem to be predictive for AL and less for infectious complications in general in ROC analysis (Figures 1, 2). One can only speculate that due to less nonspecific infectious complications, CRP course on postoperative day 4/5 seems to be a more sensitive marker for the occurrence of AL. In a meta-analysis, Paradis et al. also investigated the diagnostic characteristics of CRP levels between postoperative days 3–5 (8). Overall, elevated CRP levels do not prove AL, but especially further increasing CRP levels are reliable markers for potential alterations of routine postoperative course and may result in further diagnostics (8).

### Limitations

Due to the retrospective character, this study has several limitations. All patients were operated on over a period of 20 years in the same academic teaching hospital, which nonetheless is a low-volume community hospital, not expected to reach excellence. Also, due to technical improvements over time, more and more minimal invasive and robotic procedures have been performed (9, 57, 58) and neoadjuvant treatment concepts have been introduced into clinical practice (59–61). Over this time period, there have been major improvements in the perioperative management using “enhanced recovery after surgery” concepts (62, 63). Thus, we can only report on the surgical outcomes. In contrast to these expectations, the complication rates especially regarding SSI, AL, and mortality in this retrospective analysis of routine use of antibiotic decontamination in gastrointestinal surgery compare very well with the results of cancer surgery in currently published studies, reviews, and meta-analyses (4, 5, 9, 28, 30, 49).

Furthermore, one must assume that minor complications (Dindo–Clavien grade I–II) may be rather underrepresented. However, major complications with the need for interventional or surgical reintervention (Clavien–Dindo IIIa–V) are very well documented. Our data are heterogeneous as we report all cases using a perioperative antibiotic decontamination regimen representing high-risk anastomosis in gastric, sigmoid, and rectal cancer surgery. Our data lack a control group because in our center nearly all patients are on treatment using the SDD or modified SDD regimen. However, otherwise, a lot of outcome data and some comparable outcome data using similar SDD regimens are available in the literature for comparison (4, 5, 9, 13, 16, 17, 48, 49).

## Conclusion

The concept of perioperative antibiotic bowel decontamination in gastrointestinal surgery based on the use of a (modified) SDD regimen may be able to improve patient safety and surgical outcome in gastrointestinal oncologic surgery in a routine clinical setting. Based on new experimental data, agents other than antibiotics, such as polyphosphates or protease inhibitors, may be an alternative in the future but have not yet been introduced into clinical practice (64, 65).

## Data Availability

The raw data supporting the conclusions of this article will be made available by the authors without undue reservation.
